# Supplementary material for: Automatic speech recognition predicts contemporaneous earthquake fault displacement
Source: Nat Commun. 2025 Jan 27;16:1069. doi: 10.1038/s41467-025-55994-9 (PMC11772777; doi:10.1038/s41467-025-55994-9)
Supplement: Supplementary file 1 — Supplementary Information [file 41467_2025_55994_MOESM1_ESM.pdf]

Supplementary Information for "Automatic Speech Recognition Predicts Contemporaneous Earthquake Fault Displacement"

Christopher W. Johnson[1*†], Kun Wang[1,2†] and Paul A. Johnson[1]

[1]Los Alamos National Laboratory, EES-17 National Security Earth Science, Los Alamos, New Mexico, 87545, USA .
[2]now at ExxonMobil Technology and Engineering Company, Energy Sciences Research Division, New Jersey, USA .

*Corresponding author(s). E-mail(s): cwj@lanl.gov;
†Authors contributed equally to this work.

This Supplementary Information includes Figures S1-S3.

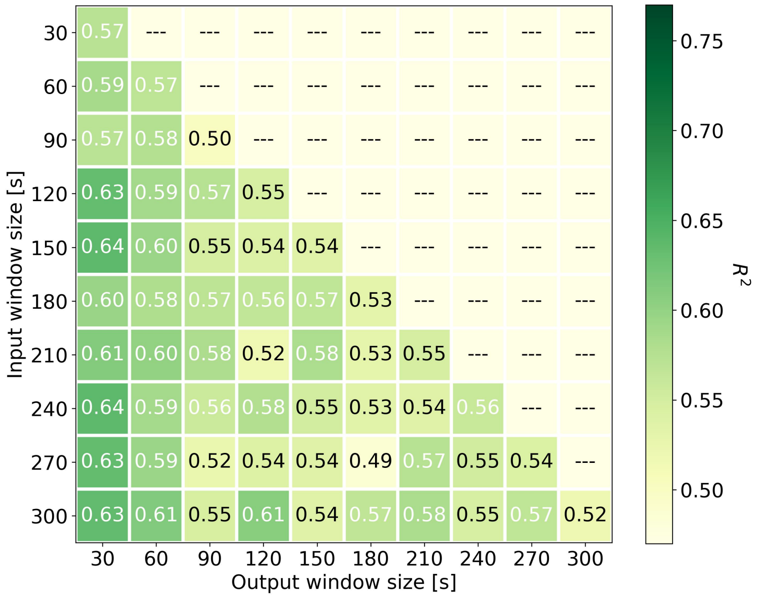

Supplementary Fig. S1 Grid search for different seismic-data input and displacement-prediction window lengths, showing R^2 of the contemporaneous predictions.

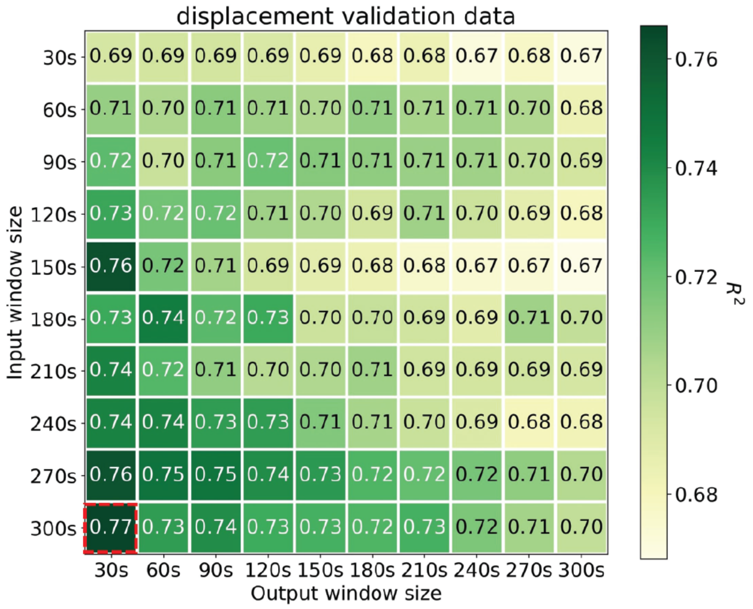

Supplementary Fig. S2 Grid search for different seismic-data input and displacement-prediction window lengths, showing R^2 of the future predictions.

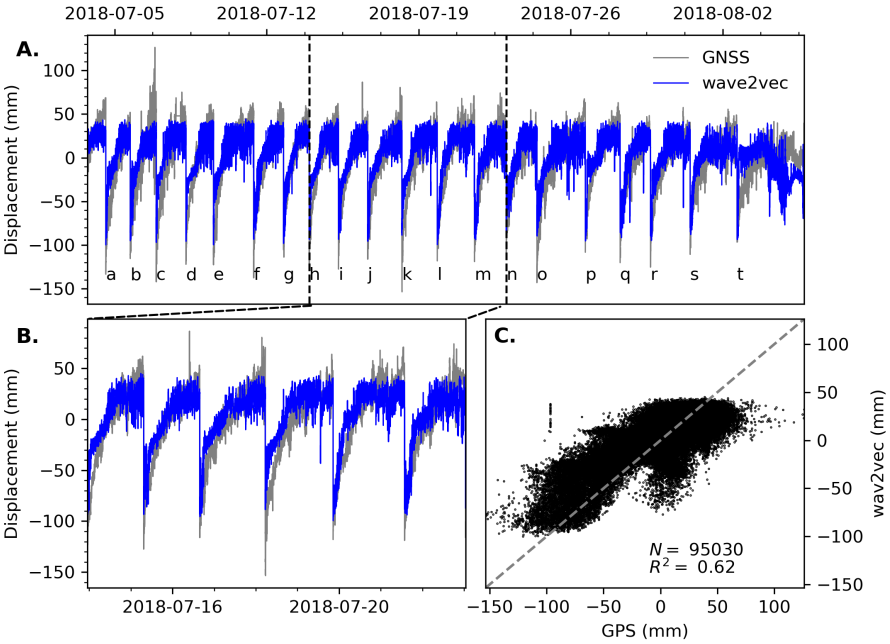

Supplementary Fig. S3 Future displacement prediction. Figure layout is the same as Fig. 3 in main text.
